# Risk factors associated to a high *Mycobacterium tuberculosis* complex seroprevalence in wild boar (*Sus scrofa*) from a low bovine tuberculosis prevalence area

**DOI:** 10.1371/journal.pone.0231559

**Published:** 2020-04-17

**Authors:** Lucía Varela-Castro, Vega Alvarez, Iker A. Sevilla, Marta Barral

**Affiliations:** Animal Health Department, NEIKER-Instituto Vasco de Investigación y Desarrollo Agrario, Derio, Bizkaia, Spain; Universitat Autonoma de Barcelona, SPAIN

## Abstract

Animal tuberculosis is a worldwide zoonotic disease caused principally by *Mycobacterium bovis*, a member of the *Mycobacterium tuberculosis complex* (MTC). In southern Iberian Peninsula, wild reservoirs such as the wild boar, among other factors, have prevented the eradication of bovine tuberculosis. However, most of the studies have been focused on south-central Spain, where the prevalence of tuberculosis is high among wild ungulates and cattle herds. In northern regions, where wild boar density and bovine tuberculosis prevalence are lower, fewer studies have been carried out and the role of this species is still under debate. The aim of this study was to describe the temporal and spatial distribution of antibodies against MTC in wild boar from the Basque Country, northern Spain. Sera from 1902 animals were collected between 2010 and 2016. The seroprevalence was determined with an in house enzyme-linked immunosorbent assay and the search of risk factors was assessed by Generalized Linear Models. Overall, 17% of wild boars (326/1902; 95%CI, [15.5%–18.9%]) showed antibodies against MTC. Risk factors associated with seropositivity were the year and location of sampling, the number of MTC positive cattle, the distance to positive farms and the percentage of shrub cover. Younger age classes were associated with increased antibody titres among seropositive individuals. The seroprevalence detected was higher than those previously reported in neighbouring regions. Hence, further studies are needed to better understand the role of wild boar in the epidemiology of tuberculosis in low tuberculosis prevalence areas and consequently, its relevance when developing control strategies.

## Introduction

Animal tuberculosis (TB) is a worldwide zoonotic disease caused principally by *Mycobacterium bovis*, a member of the *Mycobacterium tuberculosis* complex (MTC) that infects a wide range of domestic and wildlife species [1]. Because of its impact on public health and economic losses in livestock industry, eradication programs in cattle have been implemented in Europe through the last decades [2]. Meanwhile, the increase of wild ungulates populations reported in Europe results in biodiversity reduction and the increment of competent hosts for many diseases, including animal TB [3–5]. This change comes partially from the absence of predators, which could potentially contribute to both wild ungulates populations and diseases control. The appearance of habitats suitable for wild ungulates due to increased food availability and rural abandonment may also favour this tendency [4–6]. Thus, the implication of wild reservoirs, among other factors, has prevented the complete eradication of bovine TB in many countries [7]. Some recognized examples are the Eurasian wild boar (*Sus scrofa*) and the red deer (*Cervus elaphus*) in the Iberian Peninsula [2]. Moreover, other ungulates and carnivores seem to play a role in the epidemiology of bovine TB in this territory, either as spillovers, such as the red fox (*Vulpes vulpes*), the roe deer (*Capreolus capreolus*) and the Iberian lynx (*Lynx pardinus*); or as potential reservoirs, such as the fallow deer (*Dama dama*) or the Eurasian badger (*Meles meles*) [8–12]. Together with the domestic hosts, including goats [13], sheep [14] and pigs [15], as well as the main and most well studied host, cattle [16], we are facing a multi-host pathogen system, where *M*. *bovis* persistence and transmission depends on several factors, such as the high resistance of this agent in the environment, the density of hosts and species interactions [17], a scenario most likely applicable to other members of the MTC like *M*. *caprae* and *M*. *microti*. Nevertheless, many evidences point to the wild boar as the most important wild reservoir within some Mediterranean epidemiological contexts [18], bearing in mind that domestic reservoirs (e.g. goats) might be even more relevant than this wild species [19]. Besides, its opportunistic omnivorous diet and its capacity of living in a huge variety of habitats [20] turn this ungulate into an obstacle for bovine TB control strategies when its population is infected. However, the role of this host in the epidemiology of animal TB can vary from one country to another, or even between regions of the same country, since it will not only depend on the species characteristics, but also on the environment and the probability of interacting with other susceptible individuals [21]. In the Iberian Peninsula, most of the studies performed on the epidemiology of animal TB in wild boar are focused on south-central Spain, where artificial management of game species has also increased their density and aggregation [22]. Moreover, the prevalence of TB is high among wild ungulates [11] and cattle herds [23] inhabiting this area. However, in northern Atlantic and Mediterranean regions, where wild boar density and aggregation are lower, as well as the TB prevalence among cattle herds (< 1%) [23], fewer studies have been carried out and the research related to the role of wild boar is currently ongoing [24–26]. So far, whether this wild ungulate may act as a spillover or a reservoir is still under debate in northern Spain [24,26]. Hence, an increase of research is required in order to obtain a bigger picture of the understudied low TB prevalence areas, since the relevance of wild boar may increase as the prevalence in livestock decreases [27].

Therefore, this study aimed to increase the body of knowledge on animal TB epidemiology by describing the temporal and spatial distribution of antibodies against MTC in wild boar from a low bovine TB prevalence area, as well as to identify risk factors associated to the likelihood of having contact with the bacterium.

## Materials and methods

### Ethics statement

Serum samples used in this study were obtained by competent local authorities from legally hunted wild boars or from wild boar carcasses found in the field, in complete agreement with Spanish and European regulations. No animals were killed specifically for this study. No ethical approval was deemed necessary.

### Study area

This study was carried out in the Basque Country, northern Spain. This area covers 7234 km^2^ and it is divided into three provinces (Araba, Bizkaia and Gipuzkoa), according to political and administrative criteria. In northern provinces an Atlantic climate predominates with mild winters and high precipitations. In the south, there is a Continental Mediterranean climate with hot summers and cold winters [28]. Habitats also differ, being pine forests (mainly *Pinus radiata*) more common in the north and deciduous forests (dominated by *Fagus sylvatica* and *Quercus faginea*) alternated with pastures and crops in the south. Scrublands represent almost the ten per cent of the surface of the Basque Country, being distributed throughout the whole territory [29]. The prevalence of bovine TB among cattle herds from the Basque Country was less than 0.1 per cent in 2017, remaining close to official eradication [23]. On the other hand, the management of wild boar populations in this area does not imply artificial interventions such as fencing or feeding, but mostly relies in hunting activities within certain game preserves.

### Wild boar sampling

**Fig 1:**
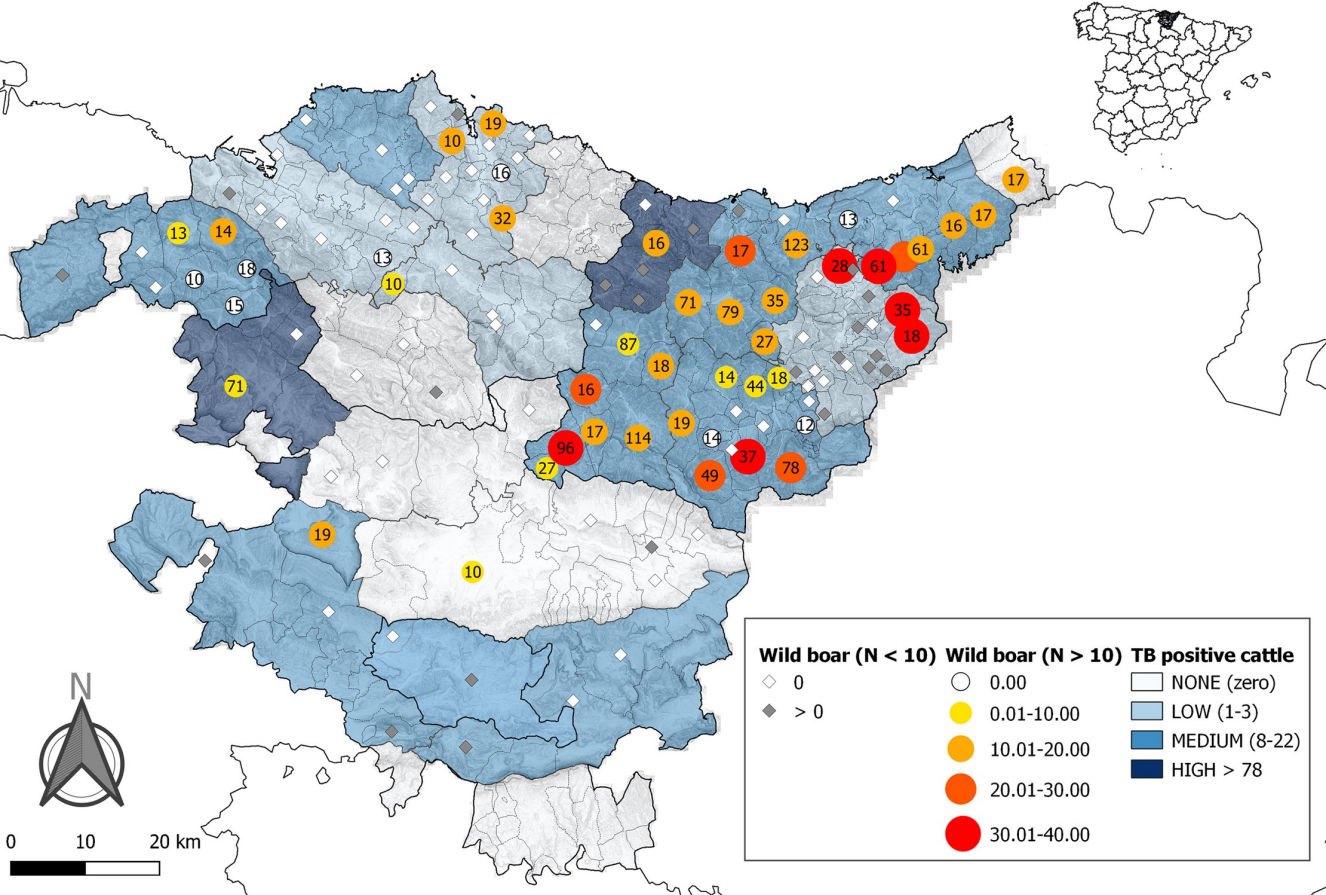
Spatial distribution of MTC seroprevalence (%) detected in wild boar and bovine TB positive cattle. Dot sizes and intensity of colour increase with the seroprevalence detected in municipalities where more than 10 wild boars were analysed. Labels inside these dots indicate the number (N) of animals analysed. Rhombuses indicate municipalities where less than 10 wild boars were analysed. Those in white mean they were negative and greys mean at least one animal was positive. Blue fill colour intensity increase with the number of TB positive cattle detected in each region: No (zero), low (1–3), medium (8–22) and high (> 78).

Serum samples from 1902 wild boars belonging to 185 out of 247 hunting areas were collected during 2010–2016 in the context of a wildlife health serological surveillance program in the Basque Country (Fig 1). Most of the animals (89.6%) were shot by authorized hunters during the regular hunting season (October to February) and sera were obtained in the field. Almost nine per cent of the serum samples were obtained from wild boar´s population control programs where animals were trapped and put down by competent authorities. A smaller proportion of sera were collected from animals with not recorded cause of death (1.5%) or from carcasses of run over animals (0.05%). Serum samples were mainly obtained by intracardiac puncture or intracavernous venipuncture, individually identified and stored at -20°C until processing.

### Serological assay

The presence of IgG antibodies against MTC was determined by using an in house enzyme-linked immunosorbent assay (ELISA) previously validated for wild boar, following the protocol previously described [30]. The control sera were the same used for the validation of this assay. All samples were analysed in duplicate. Optical densities (OD) were determined at 405 and 450 nm (MultiskanFC, ThermoScientific). OD_450 nm_ was subtracted from OD_405 nm_ and the results were expressed as an ELISA index (EI), calculating the ratio between the resulting mean sample OD and the mean OD of the positive control. Samples with an EI ≥ 0.200 were considered positive.

### Database

#### Wild boar data

Whenever it was possible, data of each wild boar such as sex (male; female), age (piglet < 1 year; yearling between 1 and 2 years; adult > 2 years), date and geographic location of collection (province, region and municipality) were recorded. Age of the animals was determined based on the sex, weight and tooth eruption patterns.

#### Livestock data

According to the last official 2009 census obtained from the Basque Statistics Institute [31], there are about 136246 cattle in 5930 farms, 272167 sheep in 4539 farms and 21547 goats in 1605 farms in the study area. Attending to these data, variables based on livestock density (number of cattle-sheep-goats/Km^2^) were calculated at region level. The whole livestock censuses of the Basque Country were taken into account for these estimations, because farms are not closed, biosafety measures are lacking and animals can remain in pastures regardless of the management system, allowing for potential direct or indirect contacts with animals outdoors, including wildlife.

#### MTC positive cattle

According to the information obtained from the Spanish Database of Animal Mycobacteriosis (mycoDB) [32], 304 MTC-infected cattle were detected by official diagnostic methods and/or inspection at slaughter and confirmed by culture in the Basque Country between September 2009 and July 2017. Herd prevalence and incidence of new positive herds during this period were highest in 2009 (0.57 and 0.55%, respectively) and lowest in 2017 (0.09 and 0.07%, respectively) according to the reports of the National Bovine TB Eradication Program [23]. Taking advantage of these data, the amount of positive cattle per region was calculated and classified according to the number of positive cows detected (zero, low, medium, high).

In addition, the Euclidean distance from each wild boar to the nearest positive cattle herd was calculated. Because of the lack of information on the exact location of each wild boar, the centroid of every municipality of sampling was used. As for the positive cows, the finest scale available was also used, being the farm´s UTM coordinates in 220 cases, the centroid of the village in 47 cases and the centroid of the municipality in 37 cases. The software QGIS Valmiera v2.2.0 [33] was used for this spatial analysis.

#### Hunted wild boar

Counts of hunted wild boar within each hunting season and game preserve were obtained from the Provincial Councils. These counts were transformed into a measure of relative abundance (hunted wild boar per km^2^) [34,35], only taking into account the habitable surface for this wild species within each game preserve, which was assessed with the software QGIS Valmiera v2.2.0 [33].

#### Vegetation cover

The vegetation cover was obtained from the 2016 Forest inventory map of the Spatial Data Infrastructure of the Basque Country [36] and from the 2006 Spanish forestry map of the Nature Databank [37]. The vegetation cover of interest was reclassified into six categories: “pine forest”, “deciduous forest”, excluding the beech forests from this category due to their lack of undergrowth; “oak forest”, “beech forest”, “scrubland” and “pastures and crops” [20,34,38]. An intersection between the surface of each municipality where every wild boar was hunted and the reclassified vegetation cover was created and the percentage of each vegetation category was calculated for every municipality. The software QGIS Valmiera v2.2.0 [33] was used for this spatial analysis.

### Statistical analysis

Two Generalized Linear Models (GLM) were implemented. The first model included 1811 wild boars and was adjusted to a binomial distribution and a logit link function, using the ELISA results (binomial variable: positive or negative) as the response variable. Then, a second model was built with a subset of positive wild boars (N = 168), using the antibody titres (continuous variable) as the response variable. This model was adjusted to a gamma distribution and a log link function. Before the implementation of these models, the normality of data was checked with the Kolgomorov-Smirnov test and several univariate analyses were performed between the response and the explanatory variables (N = 17) in order to identify potential risk factors. Non-parametric Mann-Whitney tests were used between continuous and categorical variables with two levels, while non-parametric Kruskal-Wallis tests were used when categorical variables had more than two levels; Chi-Square Tests were used between categorical variables and GLM adjusted to a gamma distribution and a log link function were used between continuous variables. In all tests, significance was set at p < 0.05. Explanatory variables for which p < 0.25 at the univariate analysis and that were correlated by less than 0.7 were considered for inclusion in the models [39]. Finally, a manual bidirectional stepwise strategy was used to select the final models. First, the two models were built including all the selected predictors. Those predictors showing a non-significant association with the response variables were sequentially excluded from each model. Confounding variables were assessed by checking for changes in the regression coefficients when removing any variable. If changes were higher than 20%, the variable was included again in the model, otherwise it was definitely removed. The Akaike Information Criteria (AIC) and the percentage of explained deviance were taken into account when selecting the final models. All the statistical analyses were performed using the R Software 3.5.0 [40]. The data set employed for the statistical analyses is deposited in a public repository [41].

## Results

Overall, 17% of wild boars (326/1902; 95%CI, [15.5%–18.9%]) showed antibodies against MTC. In Fig 1, the spatial distribution of the seroprevalence detected in wild boar among the municipalities of the Basque Country is shown, as well as the spatial distribution of the MTC-positive cattle during the same period at a regional scale. The highest seroprevalences in wild boars were mainly observed in municipalities from the east of the study area, within the province of Gipuzkoa.

**Fig 2:**
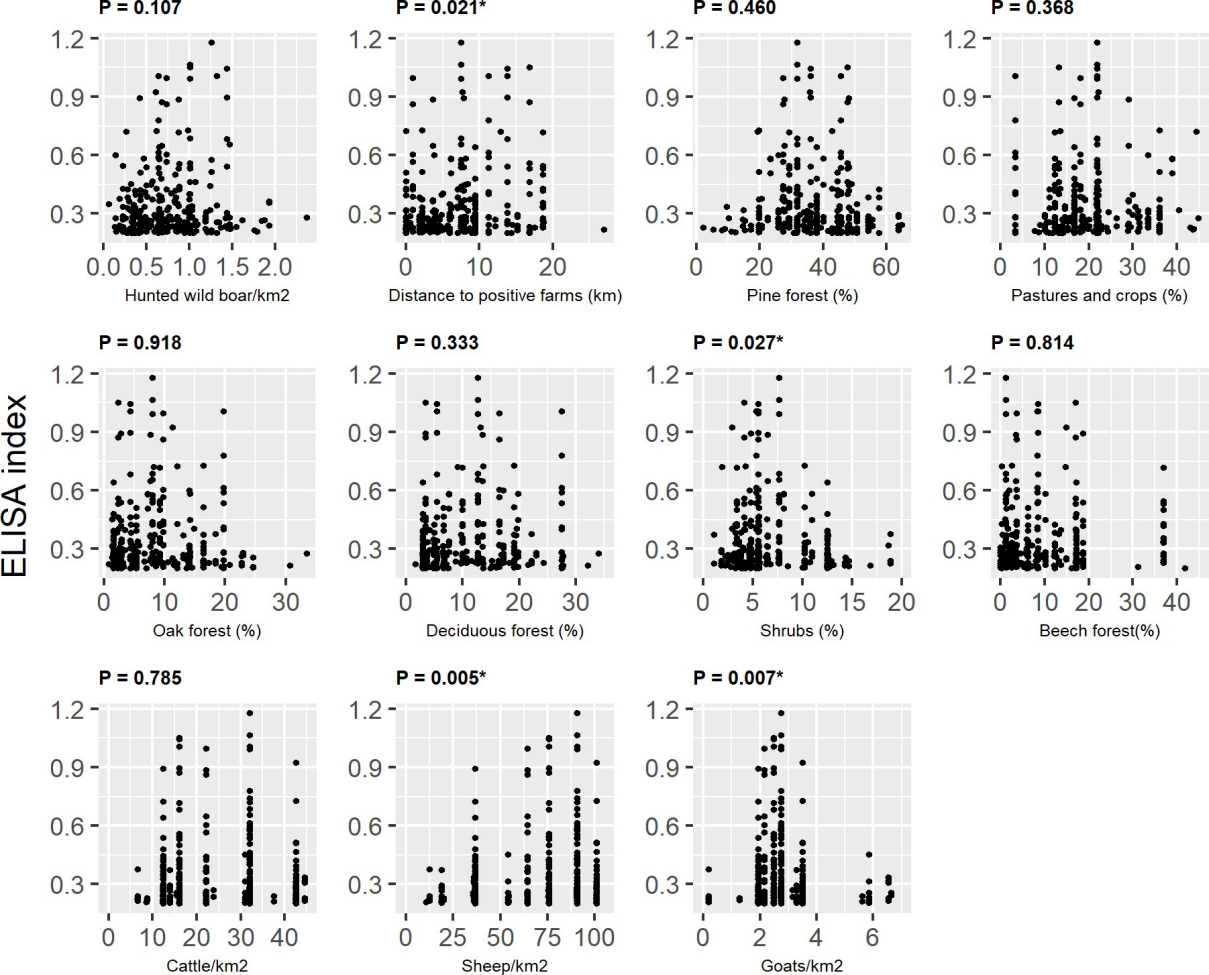
Descriptive statistics of the ELISA index of positive boars according to continuous variables. “*” indicates a significant association between the response and the explanatory variable at the univariate analysis (p <0.05). Variables with a p-value lower than 0.25 were included in the gamma model.

Results obtained after the univariate analysis are shown in Table 1 (categorical variables) and Table 2 (continuous variables) taking into account seroprevalence data (positive and negative wild boars). On the other hand, the distribution of ELISA index values among positive wild boars according to the categorical and continuous variables are described in Table 3 and Fig 2, respectively. In these four images, p-values of each univariate analysis are shown as a previous step for the selection of variables included in the binomial and gamma models.

**Table 1:** Seroprevalence of MTC detected in wild boars according to categorical variables.

Categorical variable	N. tested	% positives (95% CI)	p-value
**Sex**			0.513
Female	757	12.8 (10.6–15.4)	
Male	679	14.0 (11.6–16.8)	
**Age**			0.380
Piglet	217	15.7 (11.4–21.1)	
Yearling	438	12.1 (9.4–15.5)	
Adult	565	14.5 (11.8–17.7)	
**Sampling year**			< 0.001*
2010	138	23.2 (16.9–30.9)	
2011	190	23.2 (17.7–29.7)	
2012	128	22.7 (16.3–30.6)	
2013	320	25.0 (20.6–30.0)	
2014	571	13.3 (10.8–16.3)	
2015	323	13.0 (9.8–17.1)	
2016	232	9.9 (6.7–14.4)	
**Season**			0.330
Spring	72	13.9 (15.0–19.4)	
Summer	108	23.1 (7.7–23.7)	
Autumn	1124	17.1 (16.2–31.9)	
Winter	598	16.6 (13.8–19.7)	
**Positive cattle/region**			0.002*
Zero (0)	66	9.1 (4.2–18.4)	
Low (1–3)	392	23.2 (19.3–27.6)	
Medium (8–22)	1354	15.7 (13.8–17.7)	
High (> 78)	58	17.2 (9.6–28.9)	
**Province**			< 0.001*
Araba	94	11.7 (6.7–19.8)	
Bizkaia	297	6.7 (4.4–10.2)	
Gipuzkoa	1511	19.5 (17.6–21.6)	

“*” indicates a significant association between the response and the explanatory variable at the univariate analysis (p <0.05). P-value in bold type indicates variables included in the binomial model (after excluding correlated variables). The number of positive cattle per region was categorized as follows: zero (0), low (1–3), medium (8–22) and high (> 78).

**Table 2:** Descriptive statistics of the continuous variables values among positive and negative wild boars.

Continuous variable	ELISA POSITIVE		ELISA NEGATIVE		
	N	Median (IQR)	N	Median (IQR)	p-value
Hunted wild boar/km^2^	295	0.7 (0.5–1)	1503	0.8 (0.4–1)	**0.105**
Distance to positive cattle (km)	296	7.5 (3.3–9.4)	1515	6.1 (2.2–9.4)	**0.090**
Pine forest (%)	296	36.0 (27.8–45.7)	1515	36.0 (27.3–48.0)	0.597
Pastures & crops (%)	296	18.6 (14.6–22.2)	1515	18.6 (14.5–24.4)	0.686
Oak forest (%)	296	7.7 (2.9–12.2)	1515	5.6 (2.9–11.5)	0.789
Deciduous forest (%)	296	9.7 (3.8–16.5)	1515	7.6 (4.2–15.8)	0.676
Beech forest (%)	296	6.2 (2.5–16.9)	1515	5.0 (1.3–13.2)	0.001*
Shrubs (%)	296	5.6 (4.7–8.9)	1515	4.8 (3.8–7.7)	0.003*
Cattle/km^2^	318	22.9 (16.0–32.1)	1545	18.0 (13.8–42.6)	0.551
Sheep/km^2^	318	75.9 (56.8–90.8)	1545	64.3 (36.8–90.8)	<0.001*
Goats/km^2^	318	2.7 (2.1–2.7)	1545	2.7 (2.1–3.5)	**0.051**

“*” indicates a significant association between the response and the explanatory variable at the univariate analysis (p <0.05). P-value in bold type indicates variables included in the binomial model (after excluding correlated variables). IQR = Interquartile Range.

**Table 3:** Descriptive statistics of the ELISA index of the positive wild boars according to categorical variables.

Categorical variable	ELISA index ≥ 0.200		p-value
	N	Median (IQR)	
**Sex**			0.897
Female	97	0.267 (0.234–0.389)	
Male	95	0.274 (0.229–0.380)	
**Age**			0.001*
Piglet	34	0.284 (0.249–0.523)	
Yearling	53	0.289 (0.241–0.500)	
Adult	82	0.244 (0.217–0.313)	
**Sampling year**			**0.241**
2010	32	0.257 (0.223–0.354)	
2011	44	0.274 (0.228–0.318)	
2012	29	0.279 (0.255–0.385)	
2013	80	0.271 (0.236–0.425)	
2014	76	0.283 (0.238–0.514)	
2015	42	0.272 (0.227–0.334)	
2016	23	0.261 (0.228–0.302)	
**Season**			0.505
Spring	10	0.274 (0.229–0.291)	
Summer	25	0.271 (0.237–0.369)	
Autumn	192	0.270 (0.228–0.379)	
Winter	99	0.284 (0.239–0.395)	
**Positive cattle/region**			0.009*
Zero (0)	6	0.240 (0.230–0.254)	
Low (1–3)	91	0.376 (0.240–0.436)	
Medium (8–22)	212	0.274 (0.227–0.367)	
High (> 78)	10	0.229 (0.210–0.253)	
**Province**			0.009*
Araba	11	0.228 (0.218–0.239)	
Bizkaia	20	0.263 (0.226–0.297)	
Gipuzkoa	295	0.277 (0.234–0.400)	

“*” indicates a significant association between the response and the explanatory variable at the univariate analysis (p <0.05). P-value in bold type indicates variables included in the gamma model (after excluding correlated variables). The number of positive cattle per region was categorized as follows: zero (0), low (1–3), medium (8–22) and high (> 78).

The final (binomial) model explained 7% of the deviance (AIC = 1531.4). The results indicate that the probability of being positive for a wild boar changed over the sampling years, being significantly higher during the first years of the study period (2010–2013) when comparing with 2014 (Table 4). Hereafter, this probability began to decrease until the end of the study period, even though this change was not significant (2015–2016). As for the positive cattle, wild boars had a higher probability of being positive in regions where MTC-positive cattle were detected, compared to those where cattle were negative. Nevertheless, this increase was only significant in those regions with a low number of cattle outbreaks (Table 4). Moreover, a higher probability of being seropositive was associated with the increase of the distance to MTC positive farms (Table 4). The sampling province was also associated with the probability of being positive. This probability was higher in Araba and Gipuzkoa, compared to Bizkaia (Table 4). Lastly, a higher probability of being positive was also observed with the increase of the percentage of shrub (Table 4). With regard to the analysis of the continuous variable, the gamma model (11% of explained deviance and AIC = -176.32) showed that piglets and yearlings were significantly associated with an increase of the ELISA index, when comparing with adults (Table 4).

**Table 4:** Results of the generalized linear models.

Response variable	Predictor	Level	OR(95%CI)	Estimate	P-value
ELISA results (binomial. N = 1811)	Intercept	-	**0.01 (0.00–0.03)**	-4.68	**<0.001**
	Positive cattle/region	Zero^a^	1	NA	NA
		Low	**3.28 (1.19–9.02)**	1.19	**0.021**
		Medium	1.44 (0.54–3.85)	0.36	0.468
		High	1.64 (0.51–5.26)	0.49	0.409
	Distance to TB positive farms (km)	-	**1.04 (1.02–1.07)**	0.04	**0.002**
	Percentage of shrubs	-	**1.05 (1.01–1.09)**	0.05	**0.011**
	Year of sampling	2010	**1.79 (1.08–2.98)**	0.58	**0.025**
		2011	**2.43 (1.54–3.85)**	0.89	**<0.001**
		2012	**2.41 (1.44–4.05)**	0.88	**<0.001**
		2013	**2.12 (1.44–3.13)**	0.75	**<0.001**
		2014 ^a^	1	NA	NA
		2015	1.09 (0.71–1.67)	0.09	0.690
		2016	0.71 (0.42–1.19)	-0.34	0.196
	Provinces	Bizkaia ^a^	1	NA	NA
		Araba	**4.30 (1.66–11.12)**	1.46	**0.003**
		Gipuzkoa	**5.70 (3.28–9.93)**	1.74	**<0.001**
ELISA index (gamma. N = 168)	Intercept	-	-	-1.21	**<0.001**
	Age	Adult^a^	-	NA	NA
		Yearling	-	0.33	**<0.001**
		Piglet	-	0.27	**0.012**

Significant values are written in bold letters. NA = Not applicable.

“^a^” indicates the reference level selected for each categorical variable to run the model.

## Discussion

The research on the epidemiology of animal TB in wild boar populations is quite scarce in the north of the Iberian Peninsula when comparing to the south. For this reason, this study was necessary to obtain a wider perspective of the epidemiology of TB in wild boars from low bovine TB prevalence Atlantic areas. The ELISA test is considered a useful tool when developing a first screening in wildlife, because of its speed, ease of use and relatively low cost [42,43]. The application of this method to the 1092 wild boar sera collected in this area revealed an overall seroprevalence (17%) unexpectedly higher than that detected in neighboring regions from northern Atlantic Spain (<5%) [24,44]. This suggests that the role of wild boar in the epidemiology of TB in northern Spain may be more relevant than it was expected. Despite this, the tendency observed throughout the study period points to a general drop of the seroprevalence, even though the lowest one detected in this survey (9.9% in 2016) is still high compared to data from the aforementioned studies. Several factors may have triggered this decreasing trend in TB seroprevalence in wild boar, but it could be related to the general drop of TB herd prevalence seen in cattle during the same period (from 0.37% in 2010 to 0.17% in 2016) [23]. On the other hand, considering that in a previous study from northern Spain *M*. *avium* complex (MAC) isolates were recovered from wild boar tissues in a higher proportion than MTC isolates [24] and being aware of the antigenic repertoire similarities found between different species of this genus, some cross-reactivity with other non-tuberculous mycobacteria cannot be completely excluded. Infection with members of the MTC other than *M*. *bovis* like *M*. *caprae* or *M*. *microti* is also detectable using bPPD-based ELISAs [45]. For these reasons, further research including not only serology, but also confirmatory microbiological culture and species identification are needed to better assess the significance of different mycobacterial infections in wild boar from this region. In any case, given the high specificity attributed to this ELISA test in its validation with field samples [30], we think that the involvement of false positive results would minimally change these figures.

In the binomial model, a higher seroprevalence was found in regions where bovine outbreaks were detected, suggesting a potential risk of transmission at the wild-domestic interface. However, this increase was only significant when the amount of positive cattle was low. This could be due to the fact that interspecies interactions are not the only factor involved in the circulation and/or transmission of the bacterium. Actually, intraspecies interactions are often more common [46,47], but this is influenced by each epidemiological scenario. In our study area, most of the seropositive animals were detected in Gipuzkoa, a province where wild boars showed also the highest antibody titres. This could be due to a higher dissemination of bacteria among wild boar. Therefore, despite a bacterial circulation between cattle and wild boars cannot be dismissed, wild boar intraspecies transmission might have a more relevant role in our study area and period. However, the seroprevalences observed in some municipalities suggest that wild populations could still represent a threat in terms of TB transmission and maintenance. Thus, more studies are needed to determine the mycobacteria species and spoligotypes circulating in wild boar from this area.

Another factor significantly related to the increase of the seroprevalence was the distance between wild boars and TB positive farms. However, this association showed just the opposite effect of what was expected, since the probability of wild boars being positive increased with longer distances to the farms. Looking for a pattern at such a fine scale without the exact location of hunted animals could have led to an inaccuracy of the distance data and, consequently, to distort the statistics. Moreover, dichotomizing the ELISA index into a binomial variable results in information loss, due to the inclusion of individuals displaying an index around 0.2 (probably exposure) with those displaying an index around 1 (probably infection) in the same level. This can result in a reduced precision of the OR [48]. Despite this assumptions, a previous work found that exposure to MTC in wild boar was related to shorter distances between them and TB outbreaks in cattle, using the centroid of the commune of sampling as it was also the finest scale of spatial position available [27]. Nevertheless, the statistical approach was different, since it was carried out using a bootstrap method.

Lastly, the percentage of shrub was positively associated with the seroprevalence. Although wild boars can live in different kind of habitats, the shrub cover may be especially attractive from a survival perspective, because it can provide them a good shelter. In northern areas, unlike south-central areas in Spain [22], spatial aggregation of wildlife seems less likely to occur, since wild boar densities are lower and humid habitats prevent wild species overcrowding [24]. Thus, shrub cover may not produce a clear aggregation of wild boars, but it may hinder their movements, forcing them to use the same paths and limiting the excretion of and exposure to the bacterium to their own routes. Hence, it may not be about wild boar aggregation in northern bushy areas, but instead, we could think about a restricted movement capacity along this kind of vegetation as an enhancer of bacterial accumulation in their passages. In addition to this, shrub cover might also provide a moist microhabitat protected from the sun radiation that prompts mycobacteria survival and persistence in the environment. In the Basque Country, the shrub cover has been gradually increasing through the years, ranging from six per cent of the surface in 1986 to almost 10% during our study period [29,49]. This change in the vegetation cover seems to be linked with the abandonment of rural areas and thus, with an insufficient maintenance of forests and lands. If this rural abandonment phenomenon does not cease, other measures should be implemented to prevent the numerous problems that can derive from shrub progression, including the formation of potential hot spots for bacterial persistence.

In the gamma model, it is remarkable that, among positive wild boars, increased antibody titres were mainly observed in yearlings and piglets, compared to adult individuals. It is generally considered that increased antibody titres are associated with more severe forms of TB in many wild species, including the wild boar [50,51]. Previous studies have found evidences of severe illness in young animals, rather than in adults, as animals with large lesions in more than one anatomical region were more frequently detected among juveniles (12 to 24 months) [52], but these findings belong to a different epidemiological context (southern Spain). In another study, there was a decrease in the proportion of lesions from which mycobacteria could be isolated with increasing age [53]. The social behaviour of this wild species might also explain this difference among age classes. Adult females and their young live in groups and maintain close contact, favouring exposure by different routes. Piglets may not only suckle from their own mother, if other sows have given birth at the same time [20], increasing their chances of exposure or even of acquiring an infection. Adult males, conversely, have a solitary lifestyle, reducing their chance of contact with other wild boars out of the mating season [20] and, consequently, their risk of exposure to MTC. On the other hand, piglets and yearlings have had less time in their life to get in contact with the bacterium than the adults, and a detectable immune response needs time to develop after bacterial exposure [43]. We expected to have higher antibody titers amongst adults than amongst younger boars, as it has been suggested that recent infections in younger age-class might cause lower antibody levels and lower ELISA sensitivity [42]. In spite of this, the same study reported a seroprevalence of 29.3% (95% CI 21.3–37.2) amongst 2–6 month-old piglets with or without visible lesions and, interestingly, the antibody levels detected by the bPPD ELISAs did not correlate with the lesion score [42]. Based on the aforementioned studies, one hypothesis could be that part of adult individuals were exposed to the bacterium when they were younger but managed to control or even to clear the infection, and at the moment of hunting their immune response to an old contact or infection was less intense. Or it could be simply that reaching adulthood with progressive disease is less probable under the conditions of this area. Nevertheless, considering that the detection of higher antibody titres could be related to more extended lesions and, consequently, to higher excretion of mycobacteria [50], the dispersal behaviour of the yearlings [54] might be considered a factor that could easily contribute to the geographical spread of MTC.

The seroprevalence observed in our survey was higher than that reported earlier in other northern areas, suggesting that the spillover role of wild boar in these regions might change at any time and become more relevant, if the appropriate factors are given [26]. Hence, in areas such as the Basque Country where TB prevalence among cattle herds is minimal, a possible spillback transmission from this ungulate to cattle should not be neglected [21]. We suggest a potential risk of transmission at the wildlife-livestock interface of the study area, even though it might not be as important as the risk of wild boar or cattle intraspecies transmission. Measures to reduce the surface of shrub cover should be considered, since in addition to other risks, such as bushfires, it could be related to the exposure of wild boars to MTC. Hunting strategies should keep in mind those individuals that can have an effect on bacterial circulation or spread, such as the piglets and yearlings. The role of other domestic animals should be deeply studied, in order to gather more information of this multi-host pathogen system. Considering that the general expansion of wild boar populations in Europe through the last decades is a widely recognized problem [34,38,54], we highlight the necessity of better understanding the relevance of wild boar in the epidemiology of animal TB in northern Spain, in order to develop appropriate surveillance and control strategies, if needed, able to prevent the dissemination of the disease within wild populations and transmission to livestock.

## References

[1] Santos N, Correia-Neves M, Almeida V, Gortázar C. Wildlife Tuberculosis: A Systematic Review of the Epidemiology in Iberian Peninsula. Epidemiol Insights. 2012 10.5772/33781

[2] Gortázar C, Delahay RJ, Mcdonald RA, Boadella M, Wilson GJ, Gavier-Widen D, et al The status of tuberculosis in European wild mammals. Mamm Rev. 2012;42: 193–206. 10.1111/j.1365-2907.2011.00191.x

[3] Borowik T, Cornulier T, Jędrzejewska B. Environmental factors shaping ungulate abundances in Poland. Acta Theriol (Warsz). 2013;58: 403–413. 10.1007/s13364-013-0153-x 24244044 PMC3786087

[4] Massei G, Kindberg J, Licoppe A, Gačić D, Šprem N, Kamler J, et al Wild boar populations up, numbers of hunters down? A review of trends and implications for Europe. Pest Manag Sci. 2015;71: 492–500. 10.1002/ps.3965 25512181

[5] Lewis JS, Farnsworth ML, Burdett CL, Theobald DM, Gray M, Miller RS. Biotic and abiotic factors predicting the global distribution and population density of an invasive large mammal. Sci Rep. 2017;7: 44152 10.1038/srep44152 28276519 PMC5343451

[6] Tanner E, White A, Acevedo P, Balseiro A, Marcos J, Gortázar C. Wolves contribute to disease control in a multi-host system. Sci Rep. 2019;9: 7940 10.1038/s41598-019-44148-9 31138835 PMC6538665

[7] Schiller I, Oesch B, Vordermeier HM, Palmer M V., Harris BN, Orloski KA, et al Bovine tuberculosis: A review of current and emerging diagnostic techniques in view of their relevance for disease control and eradication. Transbound Emerg Dis. 2010;57: 205–220. 10.1111/j.1865-1682.2010.01148.x 20561288

[8] Millán J, Jiménez MÁ, Viota M, Candela MG, Peña L, León-Vizcaíno L. Disseminated Bovine Tuberculosis in a Wild Red Fox (*Vulpes vulpes*) in Southern Spain. J Wildl Dis. 2008;44: 701–706. 10.7589/0090-3558-44.3.701 18689657

[9] Balseiro A, Orusa AOR, Robetto S, Zoppi Z, Dondo A, Goria M, et al Tuberculosis in roe deer from Spain and Italy. Vet Rec. 2009;164: 468–470. 10.1136/vr.164.15.468 19363229

[10] Peña L, Garcia P, Jiménez MÁ, Benito A, Alenza MDP, Sánchez B. Histopathological and immunohistochemical findings in lymphoid tissues of the endangered Iberian lynx (*Lynx pardinus*). Comp Immunol Microbiol Infect Dis. 2006;29: 114–126. 10.1016/j.cimid.2006.01.003 16624407 PMC7136978

[11] Gortazar C, Vicente J, Boadella M, Ballesteros C, Galindo RC, Garrido J, et al Progress in the control of bovine tuberculosis in Spanish wildlife. Vet Microbiol. 2011;151: 170–178. 10.1016/j.vetmic.2011.02.041 21440387

[12] Balseiro A, Rodríguez O, González-Quirós P, Merediz I, Sevilla IA, Davé D, et al Infection of Eurasian badgers (*Meles meles*) with *Mycobacterium bovis* and *Mycobacterium avium* complex in Spain. Vet J. 2011;190: e21–e25. 10.1016/j.tvjl.2011.04.012 21612958

[13] Samper S, Gavigan J, Mari A, Marti C, Garci JF. Differentiation by Molecular Typing of *Mycobacterium bovis* Strains Causing Tuberculosis in Cattle and Goats. J Clin Microbiol. 1995;33: 2953–2956. 8576352 10.1128/jcm.33.11.2953-2956.1995 PMC228613

[14] Muñoz-Mendoza M, Romero B, del Cerro A, Gortázar C, García-Marín JF, Menéndez S, et al Sheep as a Potential Source of Bovine TB: Epidemiology, Pathology and Evaluation of Diagnostic Techniques. Transbound Emerg Dis. 2015; 1–12. 10.1111/tbed.12282 25644146

[15] Parra A, Fernández-Llario P, Tato A, Larrasa J, García A, Alonso JM, et al Epidemiology of *Mycobacterium bovis* infections of pigs and wild boars using a molecular approach. Vet Microbiol. 2003;97: 123–133. 10.1016/j.vetmic.2003.08.007 14637044

[16] Cousins D V. Mycobacterium bovis infection and control in domestic livestock. OIE Rev Sci Tech. 2001;20: 71–85. 10.20506/rst.20.1.1263 11288521

[17] Renwick AR, White PCL, Bengis RG. Bovine tuberculosis in southern African wildlife: A multi-species host-pathogen system. Epidemiol Infect. 2007;135: 529–540. 10.1017/S0950268806007205 16959052 PMC2870607

[18] Naranjo V, Gortazar C, Vicente J, de la Fuente J. Evidence of the role of European wild boar as a reservoir of *Mycobacterium tuberculosis* complex. Vet Microbiol. 2008;127: 1–9. 10.1016/j.vetmic.2007.10.002 18023299

[19] Napp S, Allepuz A, Mercader I, Nofrarias M, Lopez-Soria S, Domingo M, et al Evidence of goats acting as domestic reservoirs of bovine tuberculosis. Vet Rec. 2013;172: 663 10.1136/vr.101347 23687108

[20] Yamamoto D. The Natural Boar. Wild Boar. 2007 pp. 13–33.

[21] Nugent G. Maintenance, spillover and spillback transmission of bovine tuberculosis in multi-host wildlife complexes: A New Zealand case study. Vet Microbiol. 2011;151: 34–42. 10.1016/j.vetmic.2011.02.023 21458931

[22] Vicente J, Höfle U, Garrido JM, Fernádez-De-Mera IG, Acevedo P, Juste R, et al Risk factors associated with the prevalence of tuberculosis-like lesions in fenced wild boar and red deer in south central Spain. Vet Res. 2007;38: 451–464. 10.1051/vetres:2007002 17425933

[23] Ministerio de Agricultura Pesca Alimentación y Medio Ambiente. Programa Nacional de erradicación de tuberculosis bovina presentado por España para el año 2019. 2019 [cited 26 Jun 2019] pp. 0–31. Available: https://www.mapa.gob.es/es/ganaderia/temas/sanidad-animal-higiene-ganadera/programatb2019verdefinitiva_tcm30-500265.pdf

[24] Muñoz-Mendoza M, Marreros N, Boadella M, Gortázar C, Menéndez S, de Juan L, et al Wild boar tuberculosis in Iberian Atlantic Spain: a different picture from Mediterranean habitats. BMC Vet Res. 2013;9: 176 10.1186/1746-6148-9-176 24010539 PMC3844463

[25] Gortázar C, Fernández-Calle LM, Collazos-Martínez JA, Mínguez-González O, Acevedo P. Animal tuberculosis maintenance at low abundance of suitable wildlife reservoir hosts: A case study in northern Spain. Prev Vet Med. 2017;146: 150–157. 10.1016/j.prevetmed.2017.08.009 28992920

[26] Mentaberre G, Romero B, De Juan L, Navarro-González N, Velarde R, Mateos A, et al Long-term assessment of wild boar harvesting and cattle removal for bovine tuberculosis control in free ranging populations. PLoS One. 2014;9: 1–12. 10.1371/journal.pone.0088824 24558435 PMC3928305

[27] Richomme C, Boadella M, Courcoul A, Durand B, Drapeau A, Corde Y, et al Exposure of Wild Boar to Mycobacterium tuberculosis Complex in France since 2000 Is Consistent with the Distribution of Bovine Tuberculosis Outbreaks in Cattle. PLoS One. 2013;8 10.1371/journal.pone.0077842 24167584 PMC3805591

[28] Muñoz P, Boadella M, Arnal MC, de Miguel MJ, Revilla M, Martínez D, et al Spatial distribution and risk factors of Brucellosis in Iberian wild ungulates. BMC Infect Dis. 2010;10: 46 10.1186/1471-2334-10-46 20205703 PMC2841660

[29] Departamento de Desarrollo Económico e Infraestructuras. Inventario forestal 2016–2018. Comunidad Autónoma del País Vasco. 2018 [cited 23 Sep 2019]. Available: https://www.euskadi.eus/contenidos/informacion/inventario_forestal_2016/es_agripes/adjuntos/TTHH-CAPV-COMARCAS2016.pdf

[30] Aurtenetxe O, Barral M, Vicente J, De La Fuente J, Gortázar C, Juste RA. Development and validation of an enzyme-linked immunosorbent assay for antibodies against Mycobacterium bovis in European wild boar. BMC Vet Res. 2008;4: 1–9. 10.1186/1746-6148-4-1 18976491 PMC2606677

[31] Instituto Vasco de Estadística. [cited 6 Jul 2018]. Available: http://www.eustat.eus/indice.html

[32] Rodriguez-Campos S, González S, de Juan L, Romero B, Bezos J, Casal C, et al A database for animal tuberculosis (mycoDB.es) within the context of the Spanish national programme for eradication of bovine tuberculosis. Infect Genet Evol. 2012;12: 877–882. 10.1016/j.meegid.2011.10.008 22027158

[33] Quantum GIS Development Team. Quantum GIS Geographic Information System. 2014.

[34] Acevedo P, Quirós-Fernández F, Casal J, Vicente J. Spatial distribution of wild boar population abundance: Basic information for spatial epidemiology and wildlife management. Ecol Indic. 2014;36: 594–600. Available: http://www.sciencedirect.com/science/article/pii/S1470160X13003518

[35] Keuling O, Sange M, Acevedo P, Podgorski T, Smith G, Scandura M, et al Guidance on estimation of wild boar population abundance and density: methods, challenges, possibilities. EFSA J. 2018;15: 1–48. 10.2903/sp.efsa.2018.EN-1449

[36] GeoEuskadi, Infraestructura de Datos Espaciales de Euskadi. [cited 22 Feb 2018]. Available: http://www.geo.euskadi.eus/s69-15375/es/

[37] Banco de Datos de la Naturaleza. [cited 22 Feb 2018]. Available: https://www.miteco.gob.es/es/biodiversidad/servicios/banco-datos-naturaleza/

[38] Acevedo P, Escudero MA, Muñoz R, Gortázar C. Factors affecting wild boar abundance across an environmental gradient in Spain. Acta Theriol (Warsz). 2006;51: 327–336. 10.1007/BF03192685

[39] Dormann CF, Elith J, Bacher S, Buchmann C, Carl G, Carré G, et al Collinearity: A review of methods to deal with it and a simulation study evaluating their performance. Ecography (Cop). 2013;36: 027–046. 10.1111/j.1600-0587.2012.07348.x

[40] R Development Core Team. R: A language and environment for statistical computing. 2018.

[41] Varela-Castro L, Álvarez V, Sevilla IA, Barral M. Risk factors associated to a high *Mycobacterium tuberculosis* complex seroprevalence in wild boar (*Sus scrofa*) from a low bovine tuberculosis prevalence area. Dryad Dataset. 2020 Available: 10.5061/dryad.w9ghx3fkp PMC7164644 32302328

[42] Che’ Amat A, González-Barrio D, Ortiz JA, Díez-Delgado I, Boadella M, Barasona JA, et al Testing Eurasian wild boar piglets for serum antibodies against *Mycobacterium bovis*. Prev Vet Med. 2015;121: 93–98. 10.1016/j.prevetmed.2015.05.011 26051843

[43] Pérez de Val B, Napp S, Velarde R, Lavín S, Cervera Z, Singh M, et al Serological Follow-up of Tuberculosis in a Wild Boar Population in Contact with Infected Cattle. Transbound Emerg Dis. 2017;64: 275–283. 10.1111/tbed.12368 25944524

[44] Boadella M, Acevedo P, Vicente J, Mentaberre G, Balseiro A, Arnal MC, et al Spatio-temporal trends of Iberian wild boar contact with *Mycobacterium tuberculosis* complex detected by ELISA. Ecohealth. 2011;8: 478–484. 10.1007/s10393-011-0713-y 22065174

[45] Beerli O, Blatter S, Boadella M, Schöning J, Schmitt S, Ryser-Degiorgis M-P. Towards harmonised procedures in wildlife epidemiological investigations: A serosurvey of infection with *Mycobacterium bovis* and closely related agents in wild boar (*Sus scrofa*) in Switzerland. Vet J. 2015;203: 131–133. 10.1016/j.tvjl.2014.10.023 25466577

[46] Cowie CE, Hutchings MR, Barasona JA, Gortázar C, Vicente J, White PCL. Interactions between four species in a complex wildlife: livestock disease community: implications for *Mycobacterium bovis* maintenance and transmission. Eur J Wildl Res. 2016;62: 51–64. 10.1007/s10344-015-0973-x

[47] Payne A, Philipon S, Hars J, Dufour B, Gilot-Fromont E. Wildlife Interactions on Baited Places and Waterholes in a French Area Infected by Bovine Tuberculosis. Front Vet Sci. 2017;3 10.3389/fvets.2016.00122 28138439 PMC5237639

[48] Sroka CJ, Nagaraja HN. Odds ratios from logistic, geometric, Poisson, and negative binomial regression models. BMC Med Res Methodol. 2018;18: 1–11. 10.1186/s12874-017-0458-6 30342488 PMC6195979

[49] Departamento de Desarrollo Económico e Infraestructuras. Inventario forestal 1986. Comunidad Autónoma del País Vasco. 1986 [cited 23 Sep 2019]. Available: https://www.euskadi.eus/contenidos/informacion/inventario_forestal_antiguos/es_def/adjuntos/1986 usos del suelo.pdf

[50] Chambers MA. Review of the diagnosis of tuberculosis in non-bovid wildlife species using immunological methods—An update of published work since 2009. Transboundary and Emerging Diseases. 2013 pp. 14–27. 10.1111/tbed.12094 24171845

[51] Garrido JM, Sevilla IA, Beltrán-Beck B, Minguijón E, Ballesteros C, Galindo RC, et al Protection against tuberculosis in eurasian wild boar vaccinated with heat-inactivated *Mycobacterium bovis*. PLoS One. 2011;6: 1–10. 10.1371/journal.pone.0024905 21935486 PMC3173485

[52] Martín-Hernando MP, Höfle U, Vicente J, Ruiz-Fons F, Vidal D, Barral M, et al Lesions associated with *Mycobacterium tuberculosis* complex infection in the European wild boar. Tuberculosis. 2007;87: 360–367. 10.1016/j.tube.2007.02.003 17395539

[53] Corner LA, Barrett RH, Lepper AWD, Lewis V, Pearson CW. A Survey of Mycobacteriosis of Feral Pigs in the Northern Territory. Australian Veterinary Journal. 1981 pp. 537–542. 10.1111/j.1751-0813.1981.tb00428.x 7041875

[54] Sáez-Royuela C, Tellerìa JL. The increased population of the Wild Boar (*Sus scrofa* L.) in Europe. Mamm Rev. 1986;16: 97–101. 10.1111/j.1365-2907.1986.tb00027.x

